# Inventory of the terrestrial isopods in Belgium (2011–2020)

**DOI:** 10.3897/zookeys.1101.65810

**Published:** 2022-05-18

**Authors:** Pepijn Boeraeve, Gert Arijs, Stijn Segers, Dimitri Brosens, Peter Desmet, Kristijn Swinnen, Jorg Lambrechts, Pallieter De Smedt

**Affiliations:** 1 Spinicornis, Mispeldonk 2, 2820 Bonheiden, Belgium Spinicornis Bonheiden Belgium; 2 Research Institute for Nature and Forest (INBO), Havenlaan 88 bus 73, Belgium Research Institute for Nature and Forest Havenlaan Belgium; 3 The Belgian Biodiversity Platform, WTC III, Boulevard Simon Bolivar 30, 1000 Brussels, Belgium The Belgian Biodiversity Platform Brussels Belgium; 4 Natuurpunt Studie, Coxiestraat 11, 2800 Mechelen, Belgium Natuurpunt Studie Mechelen Belgium; 5 Ghent University, Forest & Nature Lab, Geraardsbergsesteenweg 267, 9090 Gontrode (Melle), Belgium Ghent University Gontrode Belgium

**Keywords:** Detritivores, distribution, Europe, Isopoda, Oniscidea, woodlice

## Abstract

This data paper describes a recent and spatially complete inventory of the terrestrial isopods of Belgium between 2011 and 2020. During these 10 years every 10 × 10 km² cell of the Universal Transverse Mercator (UTM) grid in Belgium (373 grid cells) was visited in search for terrestrial isopods. Inventories covered different habitat types in every grid cell such as forest, wetlands or stream sides, and urban areas. Most of the dataset records were obtained by hand-collection methods such as turning stones and dead wood, or by sieving litter and through casual observations. These inventories were carried out by specialists from Spinicornis, the Belgian Terrestrial Isopod Group. Their data is complemented with pitfall trap data from scientific projects and verified citizen science data collected via waarnemingen.be and observations.be from the same time period. This resulted in 19,406 dataset records of 35 terrestrial isopod species. All dataset records are georeferenced using the centroid of their respective 5 × 5 km² UTM grid cell. The dataset is published as open data and available through the Global Biodiversity Information Facility (GBIF). Direct link to the dataset: https://doi.org/10.15468/mw9c66.

## Data published through

Boeraeve P, De Smedt P, Segers S, Arijs G, Lambrechts J, Gielen K, Swinnen K, Desmet P, Brosens D (2021). Inventory of the terrestrial isopods in Belgium (2011–2020). Version 1.8. Natuurpunt. Occurrence dataset https://doi.org/10.15468/mw9c66

## Rationale

Soils are one of the most complex and poorly studied habitats on Earth (Decaëns 2010). Soil communities are a major component of global terrestrial biodiversity, covering at least one-quarter of the world’s biodiversity (Decaëns et al. 2006) and contributing to a wide array of ecosystem functions such as nutrient cycling, carbon sequestration, plant growth, and water storage (Lavelle et al. 2006; Wall et al. 2012). Additionally, up to 80% of all terrestrial primary production directly enters the detrital food web (Moe et al. 2005) to be processed by soil organisms that can be considered as the engine of nutrient cycling worldwide. Studying soil biodiversity is challenging because of the high diversity, small size, and high abundance of the organisms (Eisenhauer et al. 2017). However, soil macro-fauna (organisms larger than 2 mm; Jefferey et al. 2010) are easier to study because of their larger size and better-known taxonomy, and soil macro-fauna can therefore serve as model organisms for soil biodiversity. An important macro-fauna group in soils are terrestrial isopods. They are highly abundant in terrestrial soils and significantly contribute to nutrient cycling (Wolters and Ekschmitt 1997; Paoletti and Hassall 1999; David and Handa 2010). At global level, terrestrial isopod diversity is poorly studied. However, the group is fairly well studied in parts of Western Europe, such as in Great Britain and Ireland (Gregory 2009) or the Netherlands (Berg et al. 2008). In Belgium, however, studies and inventories of terrestrial isopods are mainly fragmentary even though the first publications date back to the first half of the 19^th^ century and the first checklist was already made in 1870 (De Smedt et al. 2018a). A summary of the distribution of terrestrial isopods in Belgium was made around the year 2000 (Wouters et al. 2000). This summary revealed big distribution gaps for several species, and some species had been clearly overlooked. It was not until 2014 that a complete survey of the terrestrial isopods in Belgium started by a newly founded terrestrial isopod research group in Belgium called “Spinicornis” (De Smedt et al. 2018a, 2020). Spinicornis set as a goal to make an inventory of the terrestrial isopods in every 10 × 10 km² cell of the UTM (Universal Transverse Mercator) grid in Belgium (373 grid cells). During the survey, four species were newly discovered in Belgium (*Elumacaelata* (Miers, 1877), *Philosciaaffinis* Verhoeff, 1908, *Porcelliomonticola* Lereboullet, 1853, and *Trichoniscusalemannicus* Verhoeff, 1917) and an updated checklist was published (De Smedt et al. 2018a). All fieldwork was completed in 2020 and resulted in an ecological atlas (i.e., De Smedt et al. 2020) covering all native and free-ranging species. The data collected by Spinicornis was complemented with citizen-science data from the nature observations websites waarnemingen.be and observations.be, which are hosted by Stichting Natuurinformatie and managed by the nature organisations Natuurpunt and Natagora. This paper describes these data, collected between 2011 (just before Spinicornis was founded) up to and including 2020, when the atlas project was finished.

## Taxonomic coverage

The dataset covers data from 35 native species of terrestrial isopods (order Isopoda, suborder Oniscidea) found in Belgium between 2011 and 2020. Dataset records for three multispecies are also included for which species identification was not possible based on photographs or for samples lacking males (for species that can only be identified to the species level based on male genital characteristics). There are 36 native species in Belgium (De Smedt et al. 2018a) and only one native species (i.e., *Miktoniscuspatiencei* Vandel, 1946) was not detected between 2011 and 2020. Species only occurring in greenhouses were excluded, as they are not part of the native or naturalized fauna of the country. De Smedt et al. (2017) gave more information on these species and their presence in Belgium. Nomenclature follows De Smedt et al. (2018a).

### Taxonomic ranks

**Kingdom** Animalia

**Phylum** Arthropoda

**Subphylum** Crustacea

**Class** Malacostraca

**Order** Isopoda

**Suborder** Oniscidea

**Family**Armadillidiidae, Cylisticidae, Ligiidae, Oniscidae, Philosciidae, Platyarthridae, Porcellionidae, Trachelipodidae, Trichoniscidae

**Genera***Androniscus*, *Armadillidium*, *Cylisticus*, *Eluma*, *Haplophthalmus*, *Hyloniscus*, *Ligia*, *Ligidium*, *Metatrichoniscoides*, *Oniscus*, *Philoscia*, *Platyarthrus*, *Porcellio*, *Porcellionides*, *Porcellium*, *Trachelipus*, *Trichoniscoides*, *Trichoniscus*

**Species**: Table 1.

**Common names**: terrestrial isopods, woodlice

**Table 1.** Species and multispecies represented in the dataset with their number of dataset records (N_rec_), percentage of the total amount of dataset records (%), number of UTM 5 × 5 km² squares in which the species was recorded (N_SQ_) and rank (R: 1 most squares, 35: least squares).

	Total	N_rec_	(%)	NSQ	R
**Species**	**18,572**				
*Androniscusdentiger* Verhoeff, 1908		288	1.55%	128	15
*Armadillidiumalbum* Dollfus, 1887		2	0.01%	1	35
*Armadillidiumnasatum* Budde-Lund, 1885		518	2.79%	246	8
*Armadillidiumopacum* (C. Koch, 1841)		176	0.95%	86	19
*Armadillidiumpictum* Brandt, 1833		133	0.72%	58	24
*Armadillidiumpulchellum* (Zencker, 1798)		116	0.62%	57	25
*Armadillidiumvulgare* (Latreille, 1804)		1,927	10.38%	485	5
*Cylisticusconvexus* (De Geer, 1778)		39	0.21%	10	29
*Elumacaelata* (Miers, 1877)		13	0.07%	4	33
*Haplophthalmusdanicus* Budde-Lund, 1880		253	1.36%	136	14
*Haplophthalmusmengii* (Zaddach, 1844)		87	0.47%	76	20
*Haplophthalmusmontivagus* Verhoeff, 1941		179	0.96%	138	13
*Hyloniscusriparius* (C. Koch, 1838)		204	1.10%	110	16
*Ligiaoceanica* (Linnaeus, 1767)		48	0.26%	10	29
*Ligidiumhypnorum* (Cuvier, 1792)		714	3.84%	301	7
*Metatrichoniscoidesleydigii* (Weber, 1880)		27	0.15%	17	27
*Oniscusasellus* Linnaeus, 1758		3,322	17.89%	816	2
*Philosciaaffinis* Verhoeff, 1908		184	0.99%	69	22
*Philosciamuscorum* (Scopoli, 1763)		2,927	15.76%	738	3
*Platyarthrushoffmannseggii* Brandt, 1833		310	1.67%	164	9
*Porcelliodilatatus* Brandt, 1833		40	0.22%	12	28
*Porcelliolaevis* Latreille, 1804		5	0.03%	2	34
*Porcelliomonticola* Lereboullet, 1853		32	0.17%	7	31
*Porcellioscaber* Latreille, 1804		4,127	22.22%	853	1
*Porcelliospinicornis* Say, 1818		1,060	5.71%	492	4
*Porcellionidespruinosus* (Brandt, 1833)		171	0.92%	90	18
*Porcelliumconspersum* (C. Koch, 1841)		106	0.57%	62	23
*Trachelipusrathkii* (Brandt, 1833)		367	1.98%	159	10
*Trichoniscoidesalbidus* (Budde-Lund, 1880)		154	0.83%	99	17
*Trichoniscoideshelveticus* (Carl, 1908)		38	0.20%	30	26
*Trichoniscoidessarsi* Patience, 1908		95	0.51%	70	21
*Trichoniscusalemannicus* Verhoeff, 1917		18	0.10%	5	32
*Trichoniscusprovisorius* Racovitza, 1908		182	0.98%	151	11
*Trichoniscuspusillus* Brandt, 1833		516	2.78%	307	6
*Trichoniscuspygmaeus* Sars, 1898		194	1.04%	140	12
**Multispecies**	**834**				
*Haplophthalmusdanicus/mengii/montivagus*		59	7.07%	48	
*Trichoniscoidessarsi/helveticus*		44	5.28%	41	
*Trichoniscuspusillus/provisorius/alemannicus*		731	87.65%	367	
**Total**	**19,406**				

## Geographic coverage

**Figure 1.**
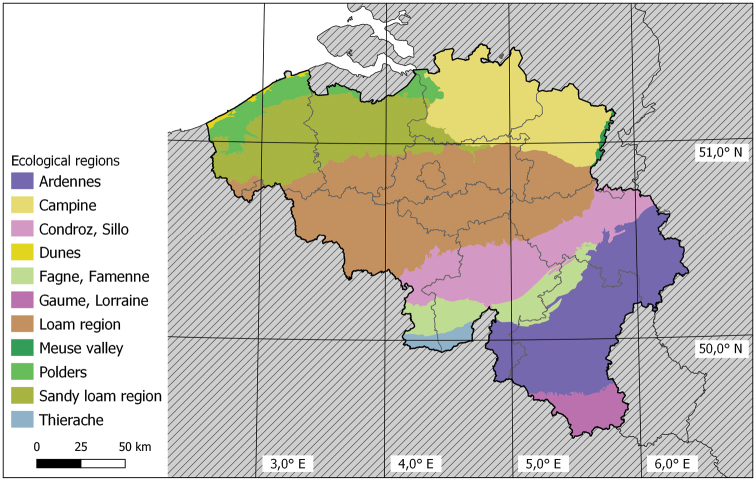
Map of Belgium indicating the different ecological regions. These ecoregions are ecologically defined areas with distinct assemblages of fauna and flora. The 11 ecological regions of Belgium are useful for analyzing terrestrial isopod diversity in Belgium (see De Smedt et al. 2020).

**Figure 2.**
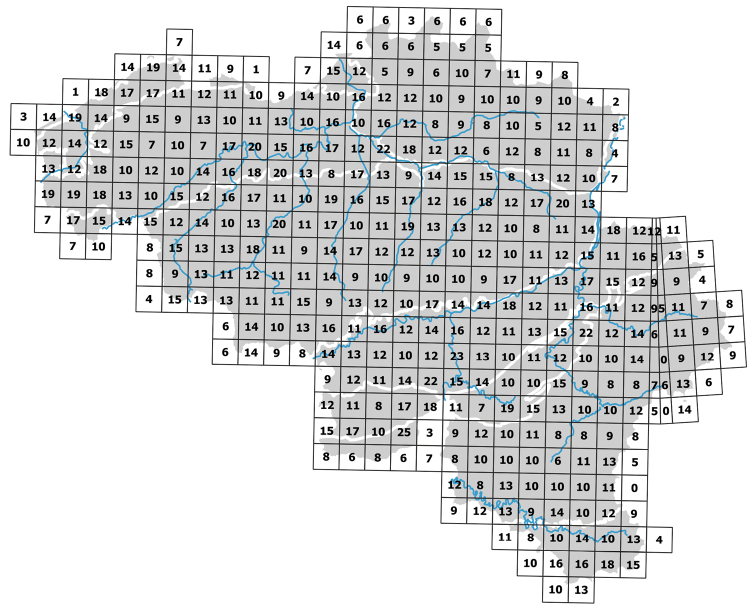
Number of terrestrial isopod species per 10 × 10 km² Universal Transverse Mercator (UTM) grid cell in Belgium.

Belgium is a small country (ca 30,500 km²) in Western Europe. It has a short shoreline (ca 65 km) along the North Sea. The shoreline borders the Netherlands in the north and France in the south. In the east, Belgium borders Germany and the Grand Duchy of Luxembourg. Belgium has a very high population density (374 inhabitants per km²). The main land use types are agriculture, urban area, and forest, with respectively 44.2%, 21.5% and 19.7% of the land area (STATBEL 2021). Despite its small size, Belgium has a rich geology ranging from a flat Atlantic region in the west, consisting of Holocene and Pleistocene deposits, to a more continental hilly landscape with Mesozoic and Pleistocene deposits in the east and the south. The Atlantic region has heavy clay soils in the northwest and loam and sandy loam soils in the central region (Fig. 1) with a high terrestrial isopod diversity (Fig. 2). On the sandy soils in the northern part of the Atlantic region, terrestrial isopod diversity is rather low (Fig. 2). Loamy soils make the connection between the Atlantic and the Continental region. The Continental region has chalky soils in the central area and in the south (Fig. 1) and has a high species richness of terrestrial isopods (Fig. 2). In between these chalky soils and in the east of the country, slate and sandstone soils are present (Fig. 1) which are more species poor (Fig. 2). Because of this rich geology, Belgium has a diverse terrestrial isopod fauna containing both Atlantic (e.g., *Trichonicoidessarsi* Patience, 1908 and *Trichonicoidesalbidus* (Budde-Lund, 1880)) and continental species (e.g., *Porcelliumconspersum* (Brandt, 1833) and *Trichoniscusalemannicus* Verhoeff, 1917).

## Bounding box

49°27'0"N and 51°32'24"N Latitude; 2°28'12"E and 6°27'36"E Longitude

## Georeferencing method

**Figure 3.**
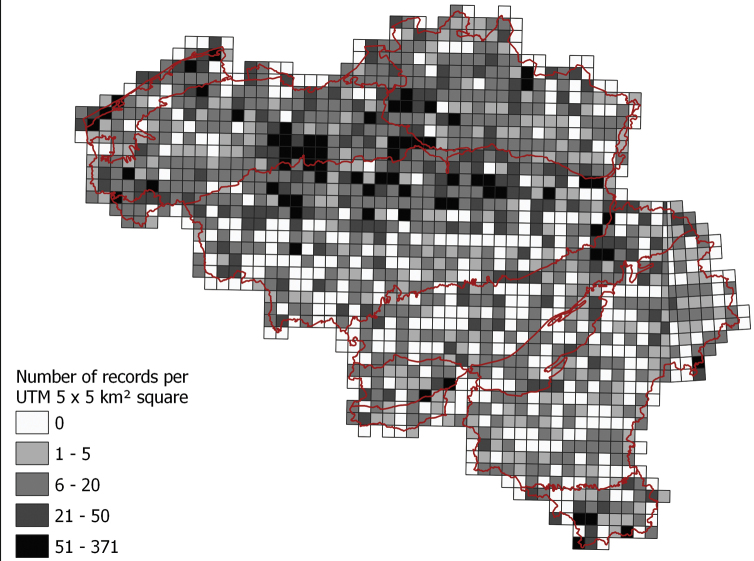
Number of dataset records per 5 × 5 km² Universal Transverse Mercator (UTM) grid cell in Belgium. Red lines delineate ecological regions.

The ObsMapp application (https://observation.org/apps/obsmapp/), developed by volunteers in collaboration with Stichting Natuurinformatie and widely used by citizen scientists in Belgium, was used to record the terrestrial isopod observations for the inventory. ObsMapp is a smartphone application where you can record and annotate observations, add pictures in the field, and make use of the GPS module in your cell phone for georeferencing (Vercayie and Herremans 2015). The complemented data from Natuurpunt and Natagora were recorded through the waarnemingen.be and observations.be websites – both using the waarnemingen.be database – or by using the apps linked to the waarnemingen.be database (ObsMapp, iObs, Obsidentify). Original point locations were recorded, and they can be requested via natuurdata@natuurpunt.be. The observation data in the waarnemingen.be database was attributed to a 10 × 10 km² grid cell for the original inventory and to a 5 × 5 km² UTM grid cell for data publication purposes. The centroids of the 5 × 5 km² grid cells were calculated using the WGS84 projection with a coordinateUncertaintyInMeters of 3,769 m. This uncertainty is the smallest circle that covers a complete UTM 5 × 5 km²-square, as data inserted in GBIF are transferred from squares to circles Wieczorek et al. 2004). The total number of dataset records per 5 × 5 km² grid cell is presented in Figure 3.

## Temporal coverage

**Figure 4.**
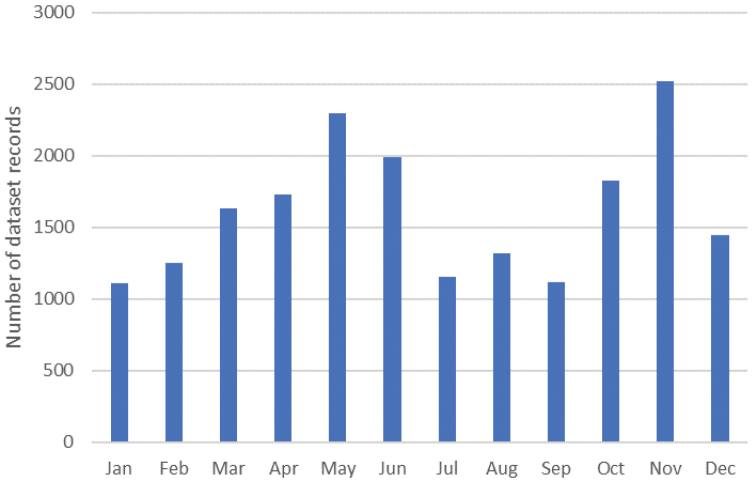
Total number of dataset records of terrestrial isopods per month across the inventory period (2011–2020).

**Figure 5.**
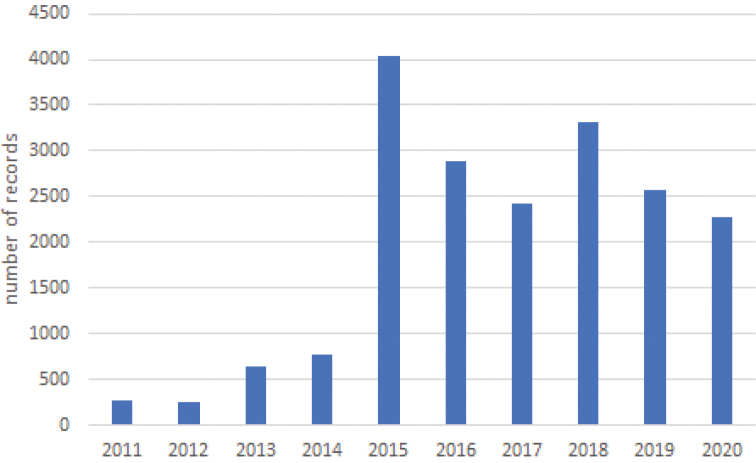
Total number of dataset records of terrestrial isopods per year across the inventory period (2011–2020).

The dataset incorporates all records from 2011 (start 2011-01-01) up to and including 2020 (end 2020-12-31): a period of exactly 10 years. Across this period, total dataset records per month ranged from 1,114 in January to 2,553 in November (Fig. 4). During the inventory period, there was a clear increase in number of dataset records since 2015, when Spinicornis was founded, with on average 2,919 (±657) dataset records per year since 2015 and only 478 (±266) dataset records per year between 2011 and 2014 (Fig. 5).

## Observers

1,275 observers contributed to the dataset, of which 606 contributed more than one dataset record and 230 contributed more than five dataset records (Table 2). This makes the number of observations per observer highly skewed (Table 2). The five observers with the largest amount of dataset records (four personal accounts and one group account), all core members of Spinicornis, represent 12,236 dataset records (or 63% of the dataset).

**Table 2.** Number of observations according to the number of users in the dataset.

Number of Observations	Number of users
1	669
2–5	376
6–10	102
11–50	104
51–200	16
201–1000	3
>1000	5

## Methodology

### Sampling methods

Terrestrial isopod distribution data was collected in three ways: i) field observations during field excursions and subsequent microscopic species identification (for smaller species), ii) pitfall trap data from scientific projects assessing terrestrial isopod community composition in different ecosystems, and iii) ad hoc citizen science observations (see below). For all dataset records, the dataset contains species ID, date, and location. Certain species can only be identified based on the male genitalia. If only females were found or the exact species could not be identified based on a photograph, the species for this record is entered as a multispecies (i.e., *Trichoniscusalemannicus/provisorius/pusillus*, *Trichoniscoideshelveticus/sarsi*, and *Haplophthalmusdanicus/mengei/montivagus*).

The field surveys consisted of monthly field excursions in search for terrestrial isopods in different habitat types through hand collection by sieving litter and topsoil, as well as by turning stones, wood, etc. The UTM 10 × 10 km² grid was chosen as survey unit, balancing between efficient time investment and necessary resolution to create representative distribution maps. This resulted in a survey of 373 grid cells. In every grid cell at least three different habitat types were visited when the habitat type was present in the grid cell, i.e., (1) a forest and if possible, an ancient deciduous forest, (2) a streamside, riverbank or (wet) grassland, and (3) an anthropogenic habitat. Old quarries and coastal habitat were also searched if present in the grid cell. Graveyards were the preferred anthropogenic habitats due to their easy access and omnipresence but also a number of public parks, (old) farms, and allotment gardens were visited. These habitat types cover the core habitat of all terrestrial isopod species in Belgium. Every grid cell was visited between September 2014 and February 2020. On every excursion at least one but mostly three or four terrestrial isopod experts from Spinicornis were present to ensure quality control of the gathered data.

Pitfall trap data from scientific projects all originated from the northern part of Belgium (Flanders). The most important projects from which data was incorporated are Nijs et al. (2016), De Smedt et al. (2018b), Pardon et al. (2019), De Smedt et al. (2021), Perring et al. (2021), and the project “Future Floodplains” (www.futurefloodplains.be). All above-mentioned projects aimed to define the terrestrial isopod community composition in (semi-) natural habitat. Specific details can be obtained in the abovementioned papers. All individuals caught during these projects were identified by terrestrial isopod experts from Spinicornis.

Ad hoc citizen science data was collected via the nature observations websites waarnemingen.be and observations.be. The experts from Spinicornis used two methods to validate ad hoc citizen science data. The first and most important method concerned validation of records for which the observer had attached clear pictures of the observed specimen. Only records with photographs enabling confirmed species identification were added to the dataset. The second method involved observations made by experienced volunteers. Dataset records were incorporated for a limited number of observers without photographs if observers made verifiable and correct identifications in the past.

### Information withheld

Location information is generalized to 5 × 5 km² UTM grid cells. Observer name, exact XY-coordinates, toponyms, and photographs are not included in the published dataset, but are known in the source database.

## Dataset

### Dataset description

Inventory of the terrestrial isopods in Belgium (2011–2020) is an occurrence dataset published by Spinicornis (Belgian Terrestrial Isopod Group), the Research Institute of Nature and Forest (INBO), and Natuurpunt Studie. The dataset represents the most complete overview of terrestrial isopods in Belgium and includes occurrences of 35 species observed between 2011 and 2020. There are 36 native terrestrial isopod species in Belgium (De Smedt et al. 2018) and only one (i.e., *Miktoniscuspatiencei* Vandel, 1946) has not been detected in Belgium between 2011 and 2020. The occurrences originate from field surveys, pitfall trap projects, and casual observations. The recorded data are registered through the citizen science portals waarnemingen.be and observations.be, which are managed by Natuurpunt Studie and Natagora, respectively. All data were verified by experts. Here, the dataset is published as a standardized Darwin Core Archive and includes for each record an occurrenceID, reference, date, location, and scientific name and, if available, also individual count, sex, lifestage, behavior, sampling protocol, and information on the identification.

The data are standardized to Darwin Core with a custom SQL view in the original waarnemingen.be database. They were published using the GBIF Integrated Publishing Toolkit (Robertson et al. 2014) instance at the INBO (https://ipt.inbo.be). The Darwin Core terms (http://rs.tdwg.org/dwc/terms/) in the dataset at the time of publication are: occurrenceID, type, language, license, rightsHolder, accessRights, references, datasetID, institutionCode, datasetName, basisOfRecord, informationWithheld, dataGeneralizations, individualCount, sex, lifestage, reproductiveCondition, behavior, occurrenceRemarks, samplingProtocol, eventDate, continent, countryCode, stateProvince, municipality, verbatimCoordinates, verbatimCoordinateSystem, verbatimSRS, decimalLatitude, decimalLongitude, geodeticDatum, coordinateUncertaintyInMeters, georeferenceRemarks, identificationVerificationStatus, identificationRemarks, taxonID, scientificName, kingdom, phylum, class, order, taxonRank, scientificNameAuthorship, vernacularName, nomenclaturalCode.

### Technical description

Source publication: https://ipt.inbo.be/resource?r=spinicornis-occurrences. This paper describes version 1.8 of this resource.

Dataset on GBIF https://www.gbif.org/dataset/b6412a28-329c-4a24-b605-bc9d1b43b5b2

DOI https://doi.org/10.15468/mw9c66

License http://creativecommons.org/publicdomain/zero/1.0/

First publication date 2021-02-24

### Usage norms

To allow anyone to use this dataset, we have released the data to the public domain under a Creative Commons Zero waiver (http://creativecommons.org/publicdomain/zero/1.0/). We would appreciate, however, that these (http://www.natuurpunt.be/normen-voor-datagebruik) norms for data use are read and followed, and that a link is provided to the original dataset (https://doi.org/10.15468/mw9c66) whenever possible. If these data are used for a scientific paper, please cite the dataset following the applicable citation norms and/or consider us for co-authorship. We are always interested in providing more information and are available for help with analysing the data for your project, so please contact us via the contact information provided in the metadata or via pepijn@spinicornis.be.
